# Methodology for Selecting Anion and Cation Exchange Membranes Based on Salt Transport Properties for Bipolar Membrane Fabrication

**DOI:** 10.1021/acsapm.5c00148

**Published:** 2025-04-17

**Authors:** Maria F. Rochow, Harrison J. Cassady, Michael A. Hickner

**Affiliations:** † Department of Material Science and Engineering, Penn State, University Park, Pennsylvania 16802-1503, United States; ‡ Department of Chemical Engineering, Penn State, University Park, Pennsylvania 16802-1503, United States

**Keywords:** bipolar membranes, ion exchange membranes, ion transport, renewable energy, electrolysis

## Abstract

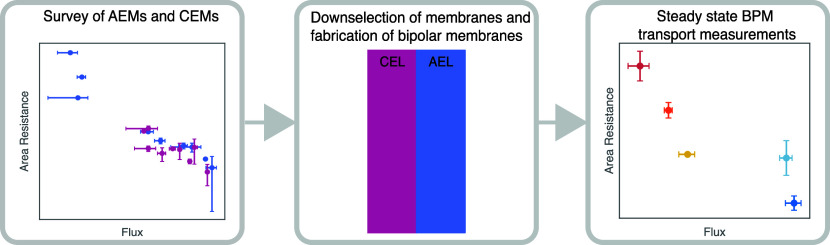

Bipolar membranes (BPMs) are a unique construction of ion exchange membranes with anion exchange and cation exchange layers in series. Due to the unique transport processes in BPMs, they are becoming an increasingly attractive option for many electrochemical devices, especially in water electrolysis and carbon dioxide reduction. However, because a large number of anion and cation exchange membranes are available, it can be difficult to select the layers for BPM fabrication, particularly when targeting specific properties for use in a device. In this study, a survey of nine anion and nine cation exchange membranes was conducted to assess their steady-state ion transport properties. The primary application of this work is seawater electrolysis; therefore, measurements of salt flux and area resistance in 0.5 mol/L sodium chloride solutions were performed. These measurements displayed a trade-off behavior, with membranes displaying higher area resistance and having a lower salt flux. Conversely, membranes with lower area resistance had a higher salt flux. From these individual membrane results, a methodology was formulated to select component membranes for BPM fabrication, primarily considering their transport characteristics. Three BPMs were fabricated using this methodology. A model was developed to integrate the parameters and ion transport properties measured from individual membranes to predict salt flux and area resistance values for a BPM. Values produced from the model were then compared with experimental salt flux and area resistance BPM measurements. Both the model and experimental salt flux and area resistance BPMs exhibited an area resistance-flux trade-off, like that of the component membranes.

## 1 Introduction

The rapid progress in developing electrochemical energy conversion and storage devices in recent decades can be attributed to the transition from conventional fossil fuels to alternative energy sources.^1^ These devices include fuel cells, electrolyzers, and redox flow batteries which are starting to see more commercial deployment.^2,3^ Many of these devices rely on ion-conducting (ion exchange) polymer membranes, which are nonporous and semipermeable separators that are selective for specific ions.^4^ A particular class of ion exchange membranes is bipolar membranes (BPMs), which have seen an increase in research interest because of their distinctive properties that provide benefits over conventional device architectures.^5^ For instance, BPMs enable stable pH gradients between electrodes, allowing flexibility in the choice of electrochemical reactions.^6−8^ These characteristics enable the possibility of alternative reactive species and electrode materials, which can influence the cost, performance, and lifespan of a device and system.^9^

Bipolar membranes are layered structures composed of a cation exchange membrane (CEM) layer and an anion exchange membrane (AEM) layer in series. These component membranes are often referred to as a cation exchange layer (CEL) and anion exchange layer (AEL).^10^ BPMs are held together at the interface of the CEL and AEL by electrostatic forces from the respective layers’ fixed charge groups. This interface is called the BPM junction and may contain an added catalyst at the junction.^11^ During water electrolysis processes that utilize BPMs, the electrolyzer operates in reverse bias where the water molecules dissociate at this junction due to the high electric field:
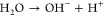
1

The hydronium ions are driven through the CEL to the cathode and the hydroxide ions are driven through the AEL to the anode.^12^

Implementation and research of BPM water electrolyzers, and more broadly, technologies using BPMs, has increased in the past few decades.^11^ However, there is still limited knowledge surrounding the fundamental transport properties of these materials.^13^ This leads to an absence of commercially available high-performance BPMs, limiting the range of membrane properties available.^14^

Given this deficiency in core understanding of BPMs, there is a gap, particularly in examining the migratory and diffusional transport components (e.g., ion crossover) of salt counter and co-ionic species, originating from the catholyte and anolyte feeds. Counterions refer to ions with a charge opposite in sign to that of the functional groups within the membrane layer, whereas co-ions possess a charge with the same sign as the functional groups. The migratory and diffusional flux mechanisms and the influence of ions from salt species on device performance have only been discussed in a few studies and mostly evaluated during electrolysis investigating ion crossover as a function of current density.^15−17^

Vermaas et al.^18^ investigated ion crossover and (photo)electrochemical water splitting performance while varying different conditions (ion concentration and type, electrolyte pH, among others). They found that for cations and anions with similar hydrated radii, cations permeated through the BPM in higher amounts than anions and that the external electrolyte salt concentration greatly influences ion crossover. Several mathematical models have been proposed that describe ion transport in BPMs, however, most of these model the membrane potential under specific conditions, use parameters that are difficult to measure experimentally, or focus solely on the ion transport of hydroxide and hydronium species.^13,19−22^

Survey studies are integral for helping to predict the performance of ion exchange membranes in electrochemical devices and understanding the fundamental transport properties across a wide range of materials. Espinoza et al.^23^ examined 40 different commercial ion exchange membranes at high salinities for their counterion conductivity and counterion/co-ion selectivity trade-off behavior, in addition to partition and diffusion selectivity. Since there are only a few commercial BPMs available, the work reported in this paper serves as a starting point for identifying ion exchange layers for bipolar membrane fabrication and correlating monopolar membrane transport measurements to bipolar membrane transport.

This study introduces an approach for selecting component membranes that circumvents the need for extensive BPM device testing, specifically targeting seawater electrolysis applications. A comprehensive survey of commercially available CEMs and AEMs was conducted. Furthermore, a new metric for membrane evaluation was established to guide subsequent BPM fabrication. This research provides fundamental insight into the steady-state diffusion processes of sodium chloride in BPMs. Additionally, the study examines the feasibility of using measured properties for individual layers of a BPM to predict properties of the composite BPM by using a simplified model. This model, which relies on a small number of easily measurable parameters, enhances the understanding of membrane characteristics and facilitates a streamlined selection process for membrane technology applications.

## 2 Materials and Methods

### 2.1 Membranes

All membranes used in the survey were purchased from the Fuel Cell Store (Bryan, TX), except for sulfonated poly(ether sulfone) (SPES) and Nafion. Nafion was purchased from Ion Power (New Castle, DE), and SPES resin (Aquafone SES01 Series, d.f. = 0.5), was purchased from YANJIN Technology (Tianjin, China). A total of 18 commercially available membranes were selected for the survey study: nine anion exchange membranes and nine cation exchange membranes. The thicknesses of each membrane were measured in the dry state using a Mitutoyo (Kawasaki, Japan) 293-831-30 digital micrometer. Membrane thickness and ion exchange capacity values are reported in Table S1. Additionally, out of the 18 membranes surveyed, six contained reinforcement, as shown in Table S1, which details the presence and type of reinforcement. Although reinforced membranes in this study were not treated differently from nonreinforced membranes, the presence of reinforcement is recognized as a factor that may influence swelling and ion transport.^24,25^

All membranes, except for SPES, were purchased in sheet form. SPES was purchased as a raw polymer resin and prepared in-house using the following method: first, the SPES resin powder was dissolved in dimethylacetamide (DMAc) at 60 °C and a polymer mass fraction of 4%. The polymer solution was then poured onto a 9 in × 9 in borosilicate glass plate (McMaster-Carr, Elmhurst, Illinois) and dried in an oven at 80 °C for 24 h at atmospheric pressure. The glass sheet and membrane were then placed in a vacuum oven at 60 °C for 24 h to ensure complete evaporation of the DMAc. The membrane was removed from the glass plate by submerging the plate in water.^26^ However, any residual DMAc molecules remaining after the drying process will be effectively removed during the removal of the membrane from the plate in water and the subsequent ion exchange/pretreatment processes, as described below, due to their solubility in water.

Any pretreatment procedures specified by the membrane manufacturer were performed before the membrane testing. Nafion was pretreated in boiling deionized water for 1 h, which is widely considered to be the standard pretreatment procedure.^24,27^ For the membranes to be in the correct counterion form before testing, all membranes were immersed in 3 mol/L sodium chloride solution for 24 h, changing the solution three times. The membranes were then rinsed in deionized water for 24 h, with the solution changed three times to extract any remaining salt. After preparation, the membranes were stored in deionized water until use. All chemicals used were ACS reagent grade and were purchased from Sigma-Aldrich (Munich, Germany). Deionized water was produced with a Thermo Fisher Scientific (Waltham, MA) Barnstead MegaPure MP12-A.

BPMs were fabricated by rolling the CEL on top of the AEL, making sure that there were no bubbles or drops of surface water between the layers. Each of the layers was 2 × 2 cm. Since none of the measurements performed involved water splitting, no water dissociation catalyst was added to the AEL–CEL junction. The BPM was then placed and centered in between a BPM press which consisted of two machined 6 in × 6 in stainless-steel plates, with eight evenly spaced 0.25 in bolts 0.5 in from the edge. The bolts were tightened using a torque wrench (Summit Tools) to 50 in lb. The plates were then set in a 50 °C water bath for 1 h. A schematic and a diagram for the BPM press are shown in Figures S1 and S2, respectively. Following this step, the BPMs were either immediately used for an experiment or stored in deionized water. The BPM fabrication procedure was adopted based on the methodology outlined in Cassady et al.^13^

### 2.2 Salt Flux

**Figure 1:**
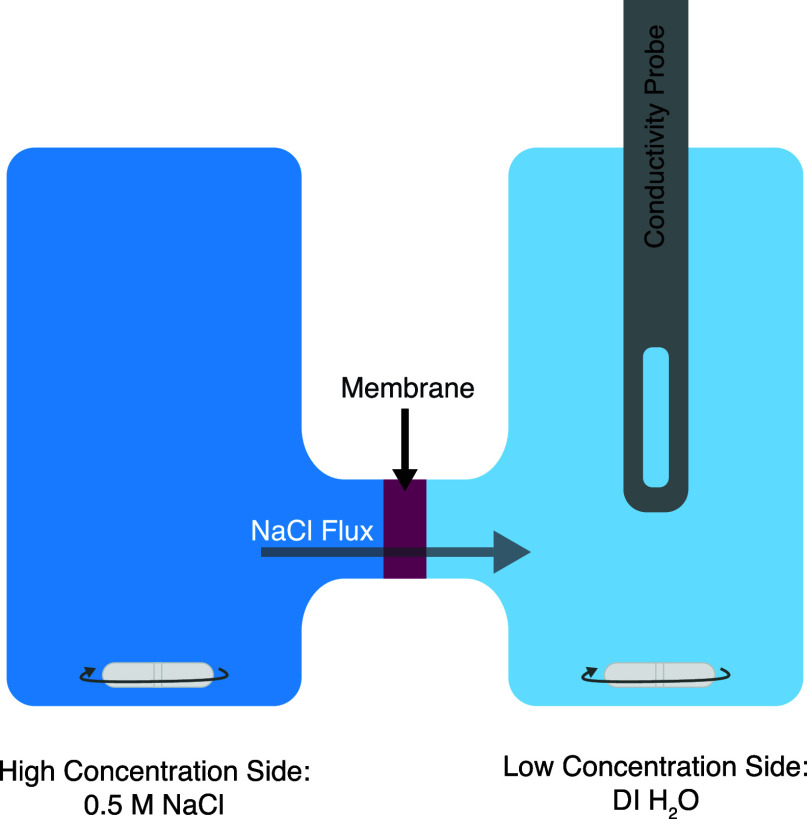
Flux experimental setup in an H-cell, where a membrane separates the high concentration side (0.5 mol/L sodium chloride) from the low concentration side (deionized water).

Salt flux was measured using an H-cell (Figure 1), with the membrane of interest separating a chamber containing a 0.5 mol/L sodium chloride solution (high concentration chamber) from a chamber containing deionized water (low concentration chamber). The area of the membrane was 197.9 mm^2^. Both chambers contained 35 mL of solution and a magnetic stir bar, which was stirred constantly for the duration of each flux measurement. The conductivity of the low-concentration chamber was monitored with an Orion DuraProbe 4-Cell conductivity probe and a Thermo Fisher Scientific (Waltham, MA) Orion Star conductivity meter.

The conductivity of the low-concentration side was monitored until a steady state was achieved. The total moles of sodium chloride transported across the membrane were then computed, calculated from the conductivity measurements, and plotted as a function of time. A linear regression was performed with the slope giving the molar flow rate across the membrane. This was divided by the membrane area to give the flux of sodium chloride through the membrane.

Flux measurements for the BPMs were measured in two orientations: with either the AEL or the CEL facing the high-concentration chamber. Triplicates of the flux measurements were taken for each orientation, after which the six measurements were averaged to give the reported salt flux value. Error bars are reported as one standard deviation from the average value.

The membrane permeability was computed from the flux with
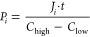
2where *P*_*i*_ is the permeability of species *i*, *J*_*i*_ is the flux of species *i*, *t* is the thickness of the membrane, *C*_high_ is the concentration of the high-concentration salt solution, and *C*_low_ is the concentration of the low-concentration salt solution. While simplified expressions for the permeability equation are commonly used to evaluate transport properties, more rigorous models have been developed to account for nonidealities.^27−30^ The diffusion coefficient was then calculated using
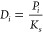
3where *D*_*i*_ is the diffusion coefficient of species *i* and *K*_*s*_ is the salt sorption coefficient.

### 2.3 Membrane Resistance

**Figure 2:**
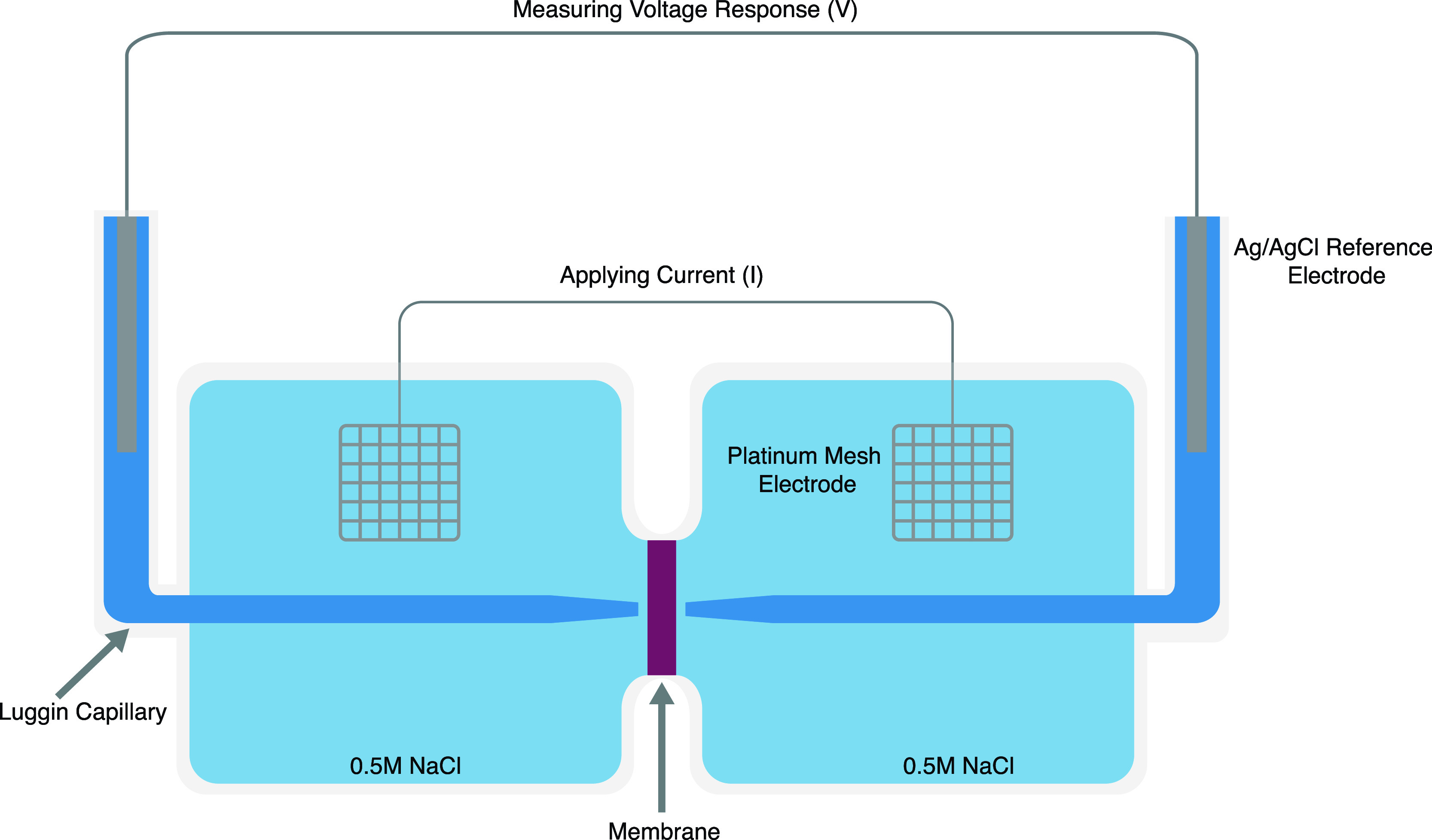
Experimental setup for a four-probe area resistance measurement.

Membrane resistance was measured by using a four-electrode setup, as shown in Figure 2. Two chambers were separated by a membrane, each chamber contained 25 mL of 0.5 mol/L sodium chloride solution. Two silver/silver-chloride reference electrodes (CHI111, CH Instruments, Austin, TX) were placed in glass Luggin capillaries. The tips of the Luggins had a diameter of 1 mm and were placed 2 mm from the surface of the membrane. A platinum mesh counter electrode was placed into each of the chambers.

Cyclic voltammetry (CV) was employed to sweep the potential between two counter electrodes by adjusting the applied current between the counter electrodes and measured using the reference electrodes. This was done using a Biologic (Seyssinet-Pariset, France) VSP-300. The sweep rate was 1000 mV/s and the potential was swept from 0 to 200 mV and then to −200 mV. An example CV plot is provided in Figure S3. Measurements on the bipolar membranes had a sweep rate of 500 mV/s and were swept from 0 to 50 mV and then to −50 mV.

A linear regression was performed on the resulting current–voltage curve, and Ohm’s Law was used to calculate the combined solution-membrane resistance. A background (solution) measurement was performed without the membrane being loaded into the cell. To calculate the membrane resistance, this background resistance was subtracted from the solution-membrane resistance measurement. The membrane resistance value is reported as the area membrane resistance, where the membrane resistance is multiplied by the surface area of the membrane. The membrane area for resistance measurements was 71 mm^2^. The resistivity, ρ, was calculated using
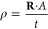
4where **R** is the membrane resistance, *A* is the membrane area, and *t* is the thickness of the membrane. Conductivity, σ, was then computed using
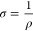
5

### 2.4 Salt and Water Sorption

Salt and water uptake were measured using a desorption method. Membrane samples were cut to 2 in × 2 in samples. The membranes were equilibrated in 0.5 mol/L sodium chloride solution for 24 h, and the solution was changed three times. Membranes were then withdrawn from the solution, and a Kimwipe was used to blot the remaining solution from the surface of the membrane. The membrane was then weighed and placed into a measured volume of deionized water (80 mL) for 24 h to desorb any sorbed salt. The conductivity of the desorption solution was measured using an Orion DuraProbe 4-Cell conductivity probe and a Thermo Fisher Scientific (Waltham, MA) Orion Star conductivity meter. The conductivity was used to compute the concentration of the desorption solution, and subsequently, the number of moles of salt desorbed from the membrane, *n̅*_*s*_. The membranes were taken out of the desorption solution, weighed to give *m*_*wet*_, and dried in a vacuum oven at 50 °C for 24 h. The membranes were then weighed to give dry mass.

The water uptake, *w*_*u*_ was calculated with^31,32^
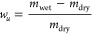
6where *m*_dry_ is the mass of the dried membrane and *m*_wet_ is the mass of the hydrated membrane. The membrane density of the dry polymer, ρ_*p*_ was calculated with
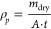
7where *A* is the membrane area and *t* is the membrane thickness. The water volume fraction, Φ_*w*_ was then calculated using
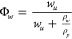
8where ρ_*p*_ is the density of the dry polymer and ρ_*w*_ is the density of pure water. The water sorption coefficient can be calculated using
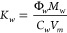
9where *M*_*w*_ is the molar mass of water, *C*_*w*_ is the concentration of water, and *V*_*m*_ is the molar volume of water.

The total amount of salt sorbed in the membrane, *n̅*_*s*_ was calculated with
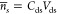
10where *C*_ds_ is the measured concentration in the desorbed solution and *V*_ds_ is the known volume of deionized water. The sorbed salt concentration in the membrane, *C̅*_*s*_, can then be calculated with
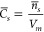
11where *V*_*m*_ is the volume of the swollen membrane. The salt sorption coefficient, *K*_*s*_, is then calculated by the equation:
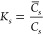
12where *C*_*s*_ is the concentration of the salt solution outside of the membrane. It should be noted that the activity coefficients are assumed to be ideal, i.e., 1, in eq 12.

Values for membrane density, water uptake, water sorption coefficient, sorbed salt concentration, and salt sorption coefficient, calculated using eqs 6–12, are reported in Table S2.

## 3 Results and Discussion

### 3.1 Down-Selection of Component Membranes for BPMs

**Figure 3:**
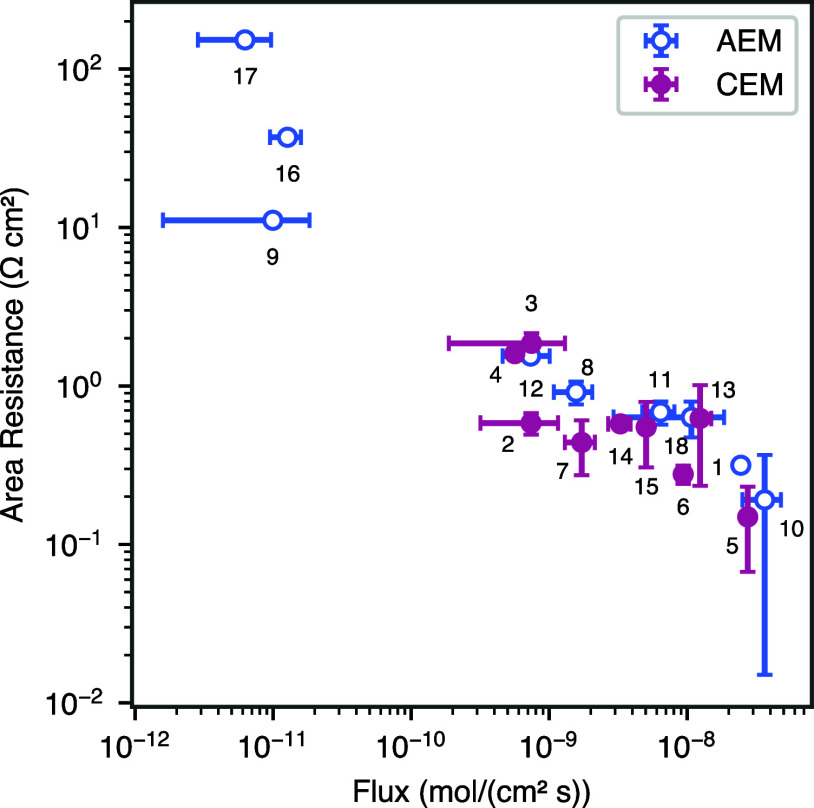
Membrane area resistance measurements in 0.5 mol/L solution versus flux of sodium and chloride ions through various CEMs and AEMs. Numerical labels correlate to specific membranes as found in Table 1.

To develop high-performing BPMs, the selection of component membranes (AEM and CEM) for the AEL and CEL is critical. Eighteen ion exchange membranes—nine AEMs and nine CEMs—were selected, and the area resistance and sodium chloride flux of these membranes were measured (Figure 3 and Table 1). Recognizing the role of membrane thickness in the device-level performance of an operating cell, the analysis was conducted to include the effects of membrane thickness in assessing transport trade-offs for these materials. The measured values for resistivity, conductivity, permeability, and diffusion coefficient which are independent of thickness are available in Table S3.

**Table 1:** Area Resistance and Flux Measurements of Individual Membranes Tested and Calculated Membrane Performance Parameter

	Membrane	Type	Flux	Area Resistance	Membrane Performance Parameter
			mol/(cm^2^ s)	(Ω cm^2^)	(s/Ω mol)
1	PiperION	AEM	2.44 × 10^–8^ ± 1.69 × 10^–9^	3.16 × 10^–1^ ± 2.05 × 10^–2^	1.30 × 10^8^ ± 1.23 × 10^7^
2	Aquivion E98-05	CEM	7.39 × 10^–10^ ± 4.21 × 10^–10^	5.83 × 10^–1^ ± 9.03 × 10^–2^	2.32 × 10^9^ ± 1.37 × 10^9^
3	Aquivion E98-15S	CEM	7.44 × 10^–10^ ± 5.57 × 10^–10^	1.86 × 10^0^ ± 2.95 × 10^–1^	7.24 × 10^8^ ± 5.53 × 10^8^
4	Fumasep FKE-50	CEM	5.63 × 10^–10^ ± 4.00 × 10^–11^	1.60 × 10^0^ ± 4.94 × 10^–2^	1.11 × 10^9^ ± 8.62 × 10^7^
5	Fumapem FS-715-RFS	CEM	2.74 × 10^–8^ ± 1.12 × 10^–9^	1.49 × 10^–1^ ± 8.20 × 10^–2^	2.45 × 10^8^ ± 1.35 × 10^8^
6	Fumapem FS-930-RFS	CEM	9.35 × 10^–9^ ± 7.64 × 10^–10^	2.77 × 10^–1^ ± 3.66 × 10^–2^	3.86 × 10^8^ ± 5.98 × 10^7^
7	Fumapem FS-930	CEM	1.72 × 10^–9^ ± 4.21 × 10^–10^	4.40 × 10^–1^ ± 1.67 × 10^–1^	1.32 × 10^9^ ± 5.97 × 10^8^
8	Fumasep FAA-3-50	AEM	1.57 × 10^–9^ ± 4.85 × 10^–10^	9.14 × 10^–1^ ± 1.50 × 10^–1^	6.98 × 10^8^ ± 2.45 × 10^8^
9	Fumasep FAB-PK-130	AEM	9.95 × 10^–12^ ± 8.37 × 10^–12^	1.11 × 10^1^ ± 5.29 × 10^–1^	9.05 × 10^9^ ± 7.62 × 10^9^
10	Fumasep FAD-55	AEM	3.64 × 10^–8^ ± 1.13 × 10^–8^	1.91 × 10^–1^ ± 1.76 × 10^–1^	1.44 × 10^8^ ± 1.40 × 10^8^
11	Fumasep FAPQ-330	AEM	6.40 × 10^–9^ ± 1.68 × 10^–9^	6.84 × 10^–1^ ± 1.14 × 10^–1^	2.28 × 10^8^ ± 7.10 × 10^7^
12	Fumasep FAS-50	AEM	7.33 × 10^–10^ ± 2.73 × 10^–10^	1.54 × 10^0^ ± 8.32 × 10^–2^	8.84 × 10^8^ ± 3.33 × 10^8^
13	Fumasep FS-720	CEM	1.24 × 10^–8^ ± 2.52 × 10^–9^	6.23 × 10^–1^ ± 3.89 × 10^–1^	1.29 × 10^8^ ± 8.49 × 10^7^
14	Nafion 212	CEM	3.28 × 10^–9^ ± 6.04 × 10^–10^	5.77 × 10^–1^ ± 2.24 × 10^–2^	5.29 × 10^8^ ± 9.97 × 10^7^
15	SPES50	CEM	5.04 × 10^–9^ ± 4.49 × 10^–10^	5.50 × 10^–1^ ± 2.43 × 10^–1^	3.61 × 10^8^ ± 1.63 × 10^8^
16	Sustainion B22-50 grade T	AEM	1.27 × 10^–11^ ± 3.21 × 10^–12^	3.71 × 10^1^ ± 1.15 × 10^0^	2.12 × 10^9^ ± 5.42 × 10^8^
17	Sustainion E28-50 grade T	AEM	6.25 × 10^–12^ ± 3.40 × 10^–12^	1.53 × 10^2^ ± 1.38 × 10^1^	1.05 × 10^9^ ± 5.78 × 10^8^
18	Sustainion X37-50 grade T	AEM	1.07 × 10^–8^ ± 7.76 × 10^–9^	6.34 × 10^–1^ ± 1.61 × 10^–1^	1.47 × 10^8^ ± 1.14 × 10^8^

For the steady-state ion flux measurement in this work, the only driving force is a concentration gradient (no electrochemical reactions). Therefore, sodium and chloride ions must transport together to satisfy electroneutrality.^33^ This means that the flux for both of these ions must be equal, and a single value for the flux is therefore reported. We have chosen to emphasize diffusive flux in this article as a proxy for what may occur under applied current in an operating cell. While this is a simplification of species crossover in electrochemical devices, the diffusion of salt species through a membrane is a reasonable place to start when selecting potential ion exchange layers for BPM fabrication.

Conversely, the sole driving force for area resistance measurements is the applied electric field, with all mobile ions (sodium and chloride) carrying the ionic current within the membrane.^34^

Both CEMs and AEMs demonstrated the characteristic flux/resistance trade-off, which is a well-documented phenomenon in multiple classes of membranes (Figure 3 and Table 1).^26,35−38^ Membranes with low salt flux had high area resistance, whereas membranes with high salt flux had low area resistance. This trade-off creates a unique optimization challenge regarding conductivity and salt crossover when selecting membranes to implement into an electrochemical device.

To establish a systematic process to down-select component membranes for BPM fabrication, a membrane performance parameter (Ξ) was defined:
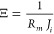
13where *R*_*m*_ is the area resistance of the membrane, and *J*_*i*_ is the flux of species, *i* through the membrane. Error for membrane performance parameter was calculated by using propagation of error.

This membrane performance parameter combines two of the most important membrane characteristics that are essential to device performance. A higher membrane performance parameter value minimizes both the area resistance and the diffusive flux through the membrane (less salt crossover). The formulation of Ξ in this work was chosen to be linear in both resistance and flux to keep the metric simple. However, more sophisticated membrane performance parameters could be devised based on the relative importance of resistance and crossover to the operating performance of the cell for a given application.

**Figure 4:**
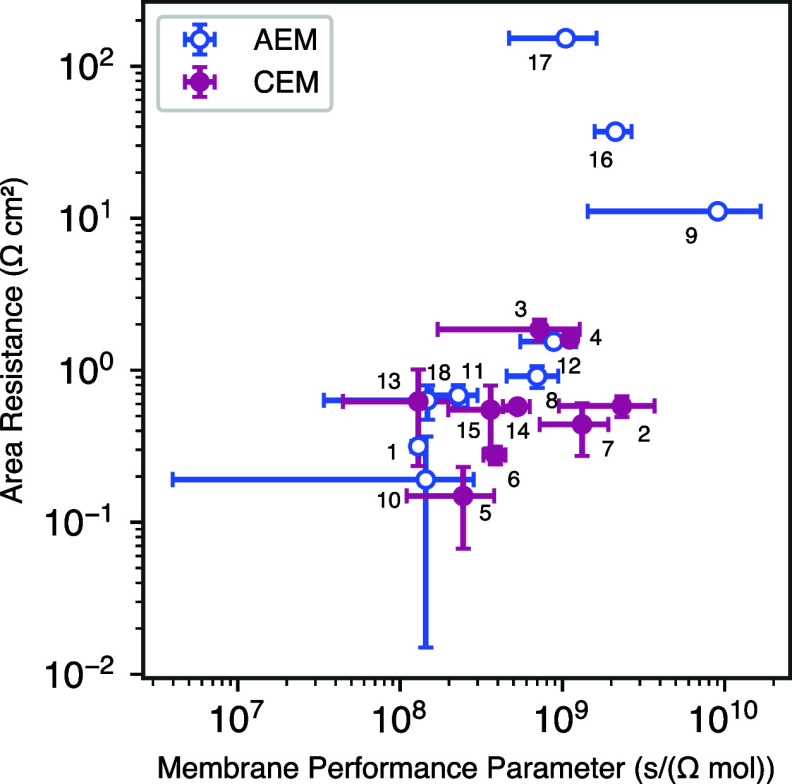
Membrane area resistance measurements in 0.5 mol/L solution versus the membrane performance parameter, defined in eq 13, of 18 CEMs and AEMs. The numerical labels correlate to the index of Table 1.

Since area resistance is particularly detrimental to many high-current devices, such as water electrolyzers, area resistance versus the membrane performance parameter was plotted to find membranes with both a high membrane performance parameter and a high area resistance value (Figure 4). Three membranes—Sustainion B28-50 grade T, Sustainion B22-50 grade T, and Fumasep FAB-PK-130—met this criterion and were not considered further due to their high resistance.

**Figure 5:**
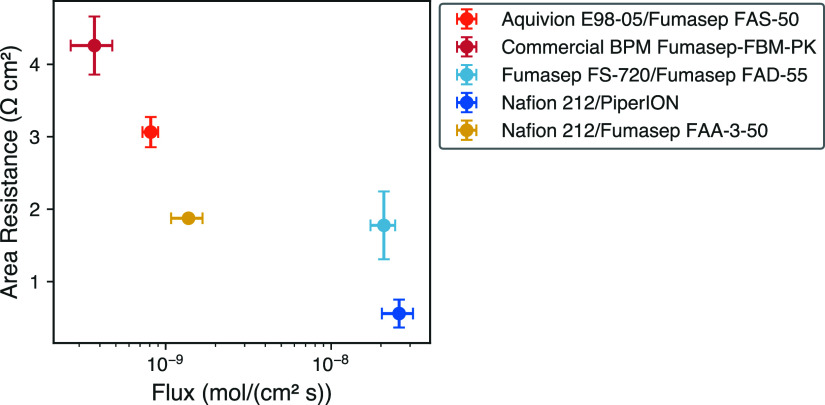
Membrane area resistance measurements in 0.5 mol/L solution versus flux of sodium and chloride ions through five different BPMs.

To demonstrate the applicability of using the membrane performance parameter metric and the component membrane properties to design a BPM, three membranes from Figure 4 were selected and fabricated into BPMs. One BPM was comprised of a CEM and AEM with a high Ξ (Aquivion E98-05 and Fumasep FAS-50), a second BPM consisted of a CEM and AEM with a midlevel Ξ (Nafion 212 and Fumasep FAA-3-50), and a third BPM consisted of a CEM and AEM with a low Ξ (Fumasep FS-720 and Fumasep FAD-55). To compare the BPMs that were selected based on the membrane performance parameter, a commercial BPM (Fumasep FBM-PK) and a BPM composed of two membranes that are individually considered standard as a cation and anion exchange membranes (Nafion 212 and PiperION) were additionally considered. The area resistance and salt flux were measured for all BPMs and are reported in Figure 5 and Table 1.

Figure 5 shows that, like the component membrane, the BPMs also exhibit the area resistance-salt flux trade-off. BPM samples with high area resistance (Aquivion E98-05/Fumasep FAS-50) demonstrated a lower salt flux. Conversely, BPMs with lower area resistance (Nafion 212/PiperION) have a higher salt flux. The highest membrane performance parameter would be found in the lower left corner of Figure 5, and the lowest membrane performance parameter would be found in the upper right corner. However, due to the general trade-off between membrane resistance and flux, membranes instead follow a trend from the upper left to the lower right. In the case of the measured membranes, the point in the upper left of this plot has the highest membrane performance parameter and the point in the lower right has the lowest membrane performance parameter. The commercial BPM (Fumasep-FBM-PK) displays the highest area resistance and lowest sodium chloride flux; this BPM is reinforced for PEEK. However, this membrane has a larger thickness, which affects both the flux and area resistance since these parameters are not thickness normalized. Two of the BPMs had the same CEM (Nafion 212/PiperION and Nafion 212/Fumasep FAA-3-50). Nafion 212/PiperION exhibited a lower flux and higher area resistance compared to those of Nafion 212/Fumasep FAA-3-50, highlighting the critical importance of AEM selection for overall ion transport.

### 3.2 BPM Steady-State Ion Transport Model

A model was developed to compare the steady-state ion transport properties of BPMs with those of their component membranes. The goal was to determine the extent to which simple, measurable properties of the component membranes could predict the ion transport behavior of the BPMs. The flux of ions in an ion exchange membrane can be described using Fick’s law of diffusion:
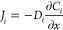
14where *J*_*i*_ is the flux of species *i*, *D*_*i*_ is the diffusion coefficient for species *i*, *C*_*i*_ is the concentration for species *i*, and *x* is the position along a membrane’s width. In examining a similar system for measuring flux in the individual membranes, a bipolar membrane separates a chamber of high-concentration *C*_high_ of salt solution (0.5 mol/L sodium chloride) and a chamber of low-concentration *C*_low_, deionized water. Using a method developed by Cassady et al.,^13^ a hypothetical salt (NaCl) concentration at the BPM junction, *C*_junction_, can be defined. The ion exchange layer of the BPM that is facing the high-concentration chamber is designated membrane A, while the layer facing the low-concentration chamber is designated membrane B. Since the sodium and chloride fluxes are stoichiometrically equivalent in this system, a single diffusion coefficient can be assigned to each specific membrane. Therefore, the diffusion coefficient for membrane A is *D*_*A*_ and that for membrane B is *D*_*B*_.

Integrating eq 14,
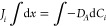
15gives:
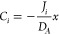
16

Applying the boundary conditions to eqs 15 and 16:
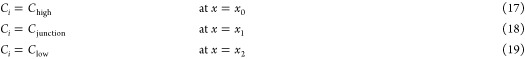
17gives:
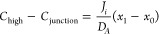
20and
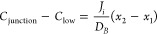
21where *x*_0_ designates the membrane-solution interface between membrane A and the high-concentration chamber, *x*_1_ is the position of the BPM junction, and *x*_2_ is the position of the membrane-solution interface between membrane B and the low-concentration chamber. This gives the thickness of membrane A as
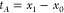
22and the thickness of membrane B as
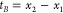
23

Combining eqs 20–23 gives the total flux through a BPM:
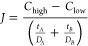
24

Bipolar membrane area resistance can be modeled after series resistors:
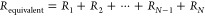
25where *R*_*N*_ is the area resistance of layer *N*. Therefore, for a bipolar membrane, the total area resistance wouldbe
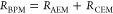
26where *R*_AEM_ and *R*_CEM_ is the resistance of the AEM and CEM, respectively.

**Figure 6:**
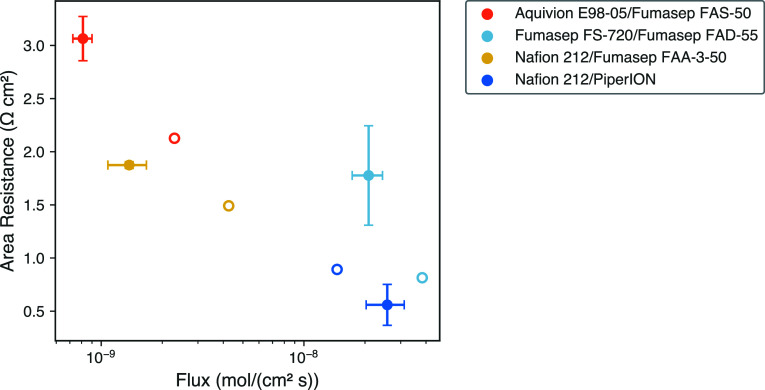
Membrane area resistance measurements in 0.5 mol/L solution vs flux of sodium and chloride ions through four different BPMs and the results of the BPM model for each of the BPMs. Values for the parameters in the BPM model were from the component membrane area resistance and flux measurements. The closed circles represent experimental measurements, while open circles are the resultant values calculated from the model. Measured and modeled values for area resistance and flux of each BPM are found in Table S4.

The results from the model demonstrate an area resistance-flux trade-off, consistent with the experimental data for both the component membranes and BPMs (Figure 6). In a comparison of the model and experimental values, in every case except for the Nafion 212/PiperION BPM, the area resistance was higher than the experimental BPMs. The model’s omission to account for any resistances stemming from the bipolar membrane junction can be attributed to these differences in resistances. According to Strathmann et al.,^39^ the bipolar membrane electrical resistance can be approximated by
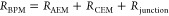
27where *R*_junction_ is the resistance of the bipolar membrane junction. *R*_junction_ was not included in the model since the resistance from the junction cannot be measured or calculated from the resistance values of the individual component membranes. Despite this limitation, considering that the individual component membrane resistances can serve as a starting point when selecting component membranes for BPM fabrication, however, the actual resistance may be higher than predicted from the component membranes alone.

Concerning the flux of sodium and chloride ions through a bipolar membrane, the model describes key features and overall trends observed in the flux experimental data; quantitative differences suggest that certain transport or interfacial phenomena may not be fully captured. This helps show that BPM fluxes can be predicted from their individual components and that there is applicability of membrane properties when going from single-layer membranes to BPMs. Furthermore, this translation of properties relies solely on simple parameters that only require the membrane(s) of interest and the salt solution. This model and selection methodology could potentially be applied to other devices that utilize BPMs that have asymmetric ion transport (mainly salt transport), especially to maximize or minimize ion crossover.

## 4 Conclusions

Eighteen commercially available cation and anion exchange membranes were surveyed for their salt transport properties by using steady-state sodium chloride flux and area resistance measurements. A membrane performance parameter was formulated to aid in the down-selection of component membranes for use as the layers in a BPM. Using the results of this survey, component membranes were selected to fabricate three bipolar membranes, for which the same transport measurements were performed.

A model was developed that integrates the component membrane and transport parameters to determine the theoretical bipolar membrane salt transport parameters. Predicted values were compared to the experimental BPM resistance and flux data. Both the experimental data and the model revealed a trade-off between area resistance and salt flux, highlighting the challenge of optimizing BPMs for applications for which minimizing salt crossover is critical. The membrane performance parameter can help screen candidate membranes that balance low salt flux and acceptable resistance.

The model reflects major characteristics and general patterns observed in the flux experimental data using simple inputs, though discrepancies occurred due to the lack of a term representing the BPM junction resistance — a property of the assembled BPM, not the individual layers. Despite this limitation, the methodology showed that component membrane properties are useful predictors of BPM performance.

This approach offers a practical starting point for selecting membranes for BPM applications, saving time and resources compared to direct BPM testing. Further research is needed to confirm its applicability to other salts and concentration conditions. Additionally, different applications may have varying requirements for the resistance and crossover. Ongoing work is integrating these BPMs into a water electrolyzer with asymmetric sodium chloride feed to assess performance and evaluate whether chloride crossover can be controlled using this membrane selection method.

## Supporting Information Available

Table of membrane properties (type, reinforcement, thickness, and ion exchange capacity), bipolar membrane press schematic, diagram of bipolar membrane press, example of a cyclic voltammetry plot, table of water uptake and salt sorption data, table of measured salt transport properties, and a table of measured and modeled salt transport parameter of the fabricated bipolar membranes (PDF)
