# Supplementary material for: Methodology for Selecting Anion and Cation Exchange Membranes Based on Salt Transport Properties for Bipolar Membrane Fabrication
Source: ACS Appl Polym Mater. 2025 Apr 17;7(9):5456–64. doi: 10.1021/acsapm.5c00148 (PMC12070371; doi:10.1021/acsapm.5c00148)
Supplement: Supplementary file 1 — ap5c00148_si_001.pdf [file ap5c00148_si_001.pdf]

Supporting Information:

A methodology for selecting anion and cation exchange membranes based on salt transport properties for bipolar membrane fabrication

Maria F. Rochow,[†,§] **Harrison J. Cassady,**[‡,‖] **and Michael A. Hickner***[,†,¶,§]

†*Department of Material Science and Engineering, Penn State, University Park, PA 16802-1503*

‡*Department of Chemical Engineering, Penn State, University Park, PA16802-1503*

¶*Department of Chemical Engineering, Penn State, University Park, PA 16802-1503*

§*Current Affiliation: Department of Chemical Engineering and Materials Science, Michigan State, East Lansing, MI 48824-1312*

‖*Current Affiliation: Energy Technologies Area, Lawrence Berkeley National Laboratory, Berkeley, CA 94720-8099*

*E-mail: mhickner@msu.edu

To provide a comprehensive understanding of the methodological framework supporting our research findings, we present a series of supplementary tables detailed below. Table S1 are the membranes used for the membrane survey and associated parameters and Table S2 calculates water uptake and salt sorption using equations found in Section 2.4 and used for calculating parameters in Table S3. Data in Table S4 is used in down-selection of component membranes and membrane performance parameter (Section 3.1), in addition to the model (Section 3.2). Table S4 is the values for Figure 5 and 6. Figures S1 and S2 show details of

membrane press for BPM fabrication. Figure S3 is an example CV plot in order to calculate area resistance.

Table S1: Membranes used for the survey.

Membrane	Type	Reinforcement[a]	Thickness[b] µm	IEC[a] meq/g
Sustainion E28-50 Grade T	AEM	Yes, PTFE	59	0.7
Fumasep FAB-PK-130	AEM	Yes, polyketone (PK)	123	0.7 - 1.0
Sustainion B22-50 Grade T	AEM	Yes, PTFE	54	0.6
Fumasep FAS-50	AEM	-	53	1.6 - 2.0
Fumasep FAA-3-50	AEM	-	32	1.6 - 2.1
Fumasep FAPQ-330	AEM	-	40	-
Sustainion X37-50 Grade T	AEM	Yes, PTFE	48	-
PiperION	AEM	-	40	2.35
Fumasep FAD-55	AEM	-	65	-
Fumasep FKE-50	CEM	-	50	1.4 - 1.5
Aquivion E98-05	CEM	-	58	> 1.0
Aquivion E98-15S	CEM	-	160	> 1.0
Fumapem FS-930	CEM	-	31	1.14
Nafion 212	CEM	-	50	0.92
SPES 50	CEM	-	21	2.08
Fumapem FS-930-RFS	CEM	Yes	30	1.15
Fumasep FS-720	CEM	-	18	1.46
Fumapem FS-715-RFS	CEM	Yes	15	1.38

[a] As listed on membrane data sheet.
[b] Measured as received in the dry membrane state.

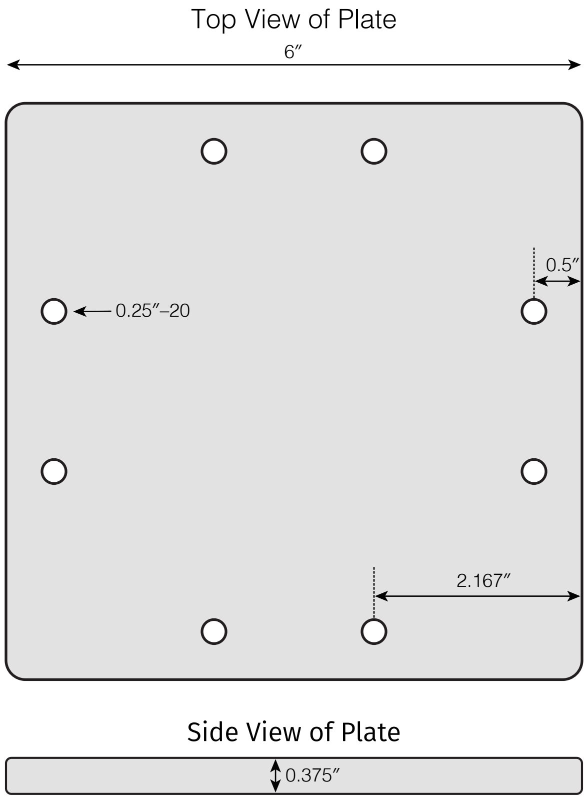

Figure S1: Membrane press schematic used for BPM fabrication.

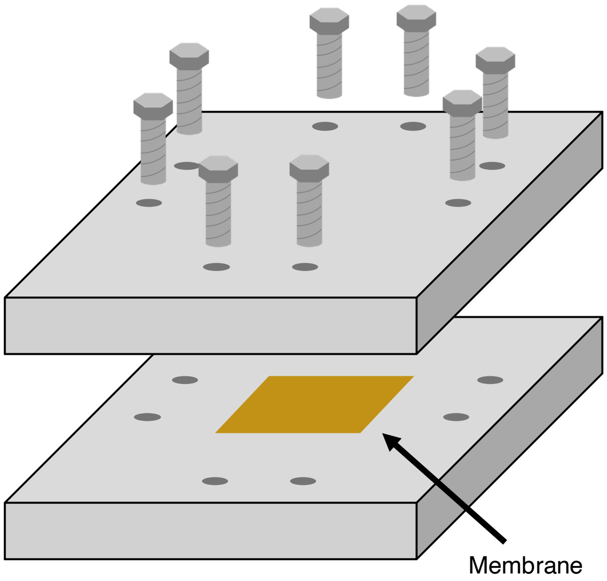

Figure S2: Diagram of membrane press used for BPM fabrication, where yellow square indicates the BPM.

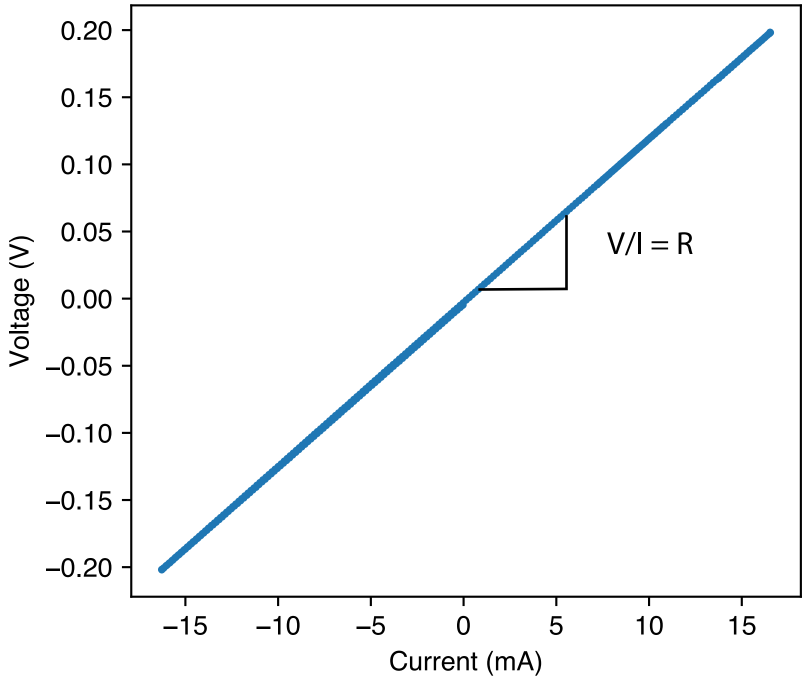

Figure S3: An example plot of the cyclic voltammetry plot for one of the membranes used in the study, Fumasep FS-720. Resistance can be calculated using the slope of a linear regression fit to the data. For a component membrane where the potential was swept from 0 mV to 200 mV and then to −200 mV at a rate of 1000 mV/s.

Table S2: Membrane water uptake and salt sorption data for 11 of the membranes in the survey.

Membrane	Membrane Density g/mL	Water Uptake	Water Sorption Coefficient	Sorbed Salt Concentration mol/L	Salt Sorption Coefficient
PiperION	1.25 ± 0.06	0.26 ± 0.06	0.19 ± 0.03	0.09 ± 0.01	0.19 ± 0.03
Aquivion E98-05	1.97 ± 0.07	0.11 ± 0.02	0.14 ± 0.05	0.02 ± 0.00	0.05 ± 0.01
Aquivion E98-15S	2.13 ± 0.04	0.16 ± 0.00	0.23 ± 0.00	0.03 ± 0.00	0.06 ± 0.00
Fumasep FKE-50	1.49 ± 0.04	0.32 ± 0.01	0.09 ± 0.00	0.04 ± 0.01	0.09 ± 0.01
Fumapem FS-715-RFS	1.56 ± 0.12	0.06 ± 0.02	0.08 ± 0.03	0.03 ± 0.00	0.07 ± 0.01
Fumapem FS-930	1.67 ± 0.04	0.09 ± 0.01	0.12 ± 0.02	0.03 ± 0.00	0.05 ± 0.00
Fumasep FAA-3-50	1.28 ± 0.03	0.29 ± 0.03	0.22 ± 0.02	0.14 ± 0.02	0.27 ± 0.05
Fumasep FAD-55	1.70 ± 0.12	0.89 ± 0.13	0.44 ± 0.04	0.28 ± 0.02	0.57 ± 0.05
Fumasep FAS-50	1.31 ± 0.03	0.29 ± 0.03	0.22 ± 0.01	0.14 ± 0.03	0.27 ± 0.05
Fumasep FS-720	1.43 ± 0.10	0.08 ± 0.05	0.05 ± 0.04	0.07 ± 0.01	0.13 ± 0.02
Nafion 212	1.98 ± 0.08	0.23 ± 0.05	0.26 ± 0.05	0.10 ± 0.00	0.20 ± 0.00

Table S3: Measured salt transport parameters for the surveyed membranes.

Membrane	Area Resistance Ω cm^2	Resistivity Ω cm	Conductivity S/cm	Flux mol/(cm^2 s)	Permeability cm^2/s	Diffusion Coefficient cm^2/s
PiperION	0.32 ± 0.02	7.90E01 ± 5	1.27E-02 ± 8E-04	2.44E-08 ± 2E-09	1.96E-07 ± 1E-08	1.04E-06 ± 7E-08
Aquivion E98-05	0.58 ± 0.09	1.17E02 ± 2E01	8.74E-03 ± 1E-03	7.39E-10 ± 4E-10	7.39E-09 ± 4E-09	1.63E-07 ± 9E-08
Aquivion E98-15S	1.86 ± 0.29	1.24E02 ± 2E01	8.22E-03 ± 1E-03	7.44E-10 ± 6E-10	2.23E-08 ± 2E-08	3.98E-07 ± 3E-07
Fumasep FKE-50	1.60 ± 0.05	3.20E02 ± 1E01	3.13E-03 ± 1E-04	5.63E-10 ± 4E-11	5.63E-09 ± 4E-10	6.55E-08 ± 5E-09
Fumapem FS-715-RFS	0.15 ± 0.08	9.94E01 ± 5E01	1.31E-02 ± 9E-03	2.74E-08 ± 1E-09	8.23E-08 ± 3E-09	1.18E-06 ± 5E-08
Fumapem FS-930-RFS	0.28 ± 0.04	9.24E01 ± 1E01	1.10E-02 ± 2E-03	9.35E-09 ± 8E-10	5.61E-08 ± 5E-09	
Fumapem-FS-930	0.44 ± 0.17	1.26E02 ± 5E01	9.15E-03 ± 4E-03	1.72E-09 ± 4E-10	1.20E-08 ± 3E-09	2.26E-07 ± 6E-08
Fumasep FAA-3-50	0.91 ± 0.15	2.86E02 ± 5E01	3.56E-03 ± 6E-04	1.57E-09 ± 5E-10	1.00E-08 ± 3E-09	3.68E-08 ± 1E-08
Fumasep FAB-PK-130	11.1 ± 0.53	9.02E02 ± 4E01	1.11E-03 ± 5E-05	9.95E-12 ± 8E-12	2.45E-10 ± 2E-10	
Fumasep FAD-55	0.19 ± 0.18	2.94E01 ± 3E01	5.27E-02 ± 3E-02	3.64E-08 ± 1E-08	4.73E-07 ± 1E-07	8.36E-07 ± 3E-07
Fumasep FAPQ-330	0.68 ± 0.11	1.71E02 ± 3E01	5.96E-03 ± 1E-03	6.40E-09 ± 2E-09	5.12E-08 ± 1E-08	
Fumasep FAS-50	1.54 ± 0.08	2.91E02 ± 2E01	3.44E-03 ± 2E-04	7.33E-10 ± 3E-10	7.77E-09 ± 3E-09	2.84E-08 ± 1E-08
Fumasep FS-720	0.62 ± 0.39	4.16E02 ± 3E02	4.25E-03 ± 4E-03	1.24E-08 ± 3E-09	3.72E-08 ± 8E-09	2.84E-07 ± 6E-08
Nafion 212	0.58 ± 0.02	1.15E02 ± 4	8.68E-03 ± 3E-04	3.28E-09 ± 6E-10	3.28E-08 ± 6E-09	1.64E-07 ± 3E-08
SPES50	0.55 ± 0.24	2.62E02 ± 1E02	4.24E-03 ± 1E-03	5.04E-09 ± 4E-10	2.12E-08 ± 2E-09	
Sustainion B22-50 Grade T	37.1 ± 1.2	6.87E03 ± 2E02	1.46E-04 ± 5E-06	1.27E-11 ± 3E-12	1.37E-10 ± 3E-11	
Sustainion E28-50 Grade T	150 ± 14	2.59E04 ± 2E03	3.88E-05 ± 3E-06	6.25E-12 ± 3E-12	7.38E-11 ± 4E-11	
Sustainion X37-50 Grade T	0.63 ± 0.16	1.32E02 ± 3E01	7.82E-03 ± 2E-03	1.07E-08 ± 8E-09	1.03E-07 ± 7E-08	

Table S4: Measured and modeled salt transport parameters for the fabricated BPMs.

	Experimental			Model		
Membrane	Flux mol/(cm^2 s)	Area Resistance Ω cm^2	Membrane Performance Parameter s/mol Ω	Flux mol/(cm^2 s)	Area Resistance Ω cm^2	Membrane Performance Parameter s/mol Ω
Nafion 212/PiperION	2.58E-09 ± 6E-9	0.56 ± 0.19	6.94E+07	1.46E-08	0.89	7.69E+07
Nafion 212/Fumasep FAA-3-50	1.37E-09 ± 3E-10	1.87 ± 0.03	3.88E+08	4.25E-09	1.49	1.58E+08
Fumasep FS-720/Fumasep FAD-55	2.08E-08 ± 3.6E-9	1.78 ± 0.47	2.70E+07	3.83E-08	0.81	3.20E+07
Aquivion E98-05/Fumasep FAS-50	8.12E-10 ± 9E-11	3.06 ± 0.21	4.02E+08	2.30E-09	2.13	2.05E+08
